# Acaricide resistance of Rhipicephalus decoloratus ticks collected from communal grazing cattle in South Africa

**DOI:** 10.5455/javar.2022.i566

**Published:** 2022-01-15

**Authors:** Mandla Yawa, Nkululeko Nyangiwe, Ishmael Festus Jaja, Munyaradzi Christopher Marufu, Charles T. Kadzere

**Affiliations:** 1 Department of Livestock and Pasture Science, University of Fort Hare, Alice, South Africa; 2 Döhne Agricultural Development Institute, Stutterheim, South Africa; 3 Department of Agriculture and Animal Health, University of South Africa, Roodepoort Johannesburg 1710, South Africa; 4 Department of Veterinary Tropical Diseases, University of Pretoria, Pretoria, South Africa

**Keywords:** Acaricide resistance, cattle, Rhipicephalus decoloratus larvae, Shaw larval immersion test

## Abstract

**Objective:**

This study aimed to determine acaricide resistance in *Rhipicephalus decoloratus* ticks collected from grazing cattle between November 2018 and May 2019 in Elundini, Senqu, and Walter Sisulu Local Municipalities in the northeastern region of the Eastern Cape Province.

**Materials and Methods:**

A sample of 20–30 adult engorged female *R.* *decoloratus* ticks were collected from at least 10 randomly selected cattle (highly tick-infested) at each dip tank and placed into the labelled plastic collection bottles containing absorbent paper and with a perforated lid at a constant room temperature of ±28°C and >70% relative humidity until resistance testing commenced. The Shaw larval immersion test method was used to determine *R. decoloratus* larvae resistance to various acaricide concentration levels [amidines, organophosphate (OP), and synthetic pyrethroids (SPs)].

**Results:**

This study found that most ticks were susceptible to exposure to different acaricide field concentrations of amidines (49% at 250 ppm), OPs (33% and 47% at 300 ppm and 500 ppm, respectively), and SPs (44% and 23% at 150 ppm and 300 ppm, respectively). The resistance testing results showed no resistance to amidines at any localities and no resistance to OP in the Senqu region. However, resistance development of the larvae to amines, OPs, and SPs was extensively observed in Senqu (18%, 6%, and 7%), Elundini (15%, 15%, and 17%), and Walter Sisulu (13%, 19%, and 9%) regions, respectively.

**Conclusions:**

The larvae’s resistance is a cause for worry. Hence, the continuous monitoring of tick resistance to commonly used acaricides will help mitigate widespread acaricidal resistance and sustain livestock productivity.

## Introduction

Among other tick species globally, *Rhipicephalus decoloratus* is the most widely distributed tick species and considered the most crucial external parasite to livestock, particularly in cattle [1]. The tickis known as the indigenous tick to the African continent and is widely distributed in tropical and subtropical regions. *Rhipicephalus decoloratus *is regarded as the major vector for transmission of tick-borne diseases, such as *Babesia bigemina*, *Anaplasma marginale*, and *Anaplasma central*, to cattle, whereas its counteracting species, *Rhipicephalus microplus*, can also transmit *Babesia bovis*, amongst other pathogens [2–4]. Heavy infestation of ticks results in the production and economic losses by reducing milk yield in cows, meat, and damage to the skin. However, over time, *R.* *decoloratus *tickshave become resistant to almost every application of registered acaricide, thus increasing its rapidity and spreading into nonendemic areas [5,6]. The tick’s lifecycle, worldwide distribution, and indiscriminate acaricide use by cattle farmers have proven to be the most significant contributing factors to the rapid establishment of tick-resistant acaricide compounds [7].

In South Africa, three synthetic acaricide chemical groups are known to be used to control ticks, including organophosphates (OPs), amidines, and pyrethroids [8,9]. Of these acaricide groups, pyrethroids and OPs are the most commonly used insecticides by most resource-limited farmers [10,11]. Sodium arsenate was the first successful acaricide used in South Africa until the first report of resistance detection on *R*. *decoloratus*. After that, benzene hexachloride (BHC) took over from arsenic as an alternative in the form of dichlorodiphenyltrichloroethane [12]. Since then, the incidence of arsenic–BHC-resistant blue ticks has increased along the country’s coastal regions [13]. In addition to this, Perez-Cogollo et al. [19] reported the first record of *R.* *microplus* being resistant to OP in the eastern regions of the Eastern Cape Province (ECP), and no reports have yet been made on the northern part of the province.

Several bioassays used in evaluating tick susceptibility to acaricide chemicals include the adult immersion test, larval packed test (LPT) [14], and larval immersion test [15]. Recently, a new bioassay method, the Larval Tarsal Test (LTT), was developed and compared to the LPT. Both tests successfully detected resistance to OP, amitraz, and coumaphos [16]. The LTT has proven to be more advantageous than the LPT since it allows a large volume of doses and compounds in a short time and fewer engorged females [17]. Tick resistance is typically established by exposing ticks to a unique type of dosage guided by the information of a susceptible reference strain; after that, a discriminating dose survival indicates acaricide resistance [17]. The objective of the current study was to determine the acaricide resistance of *R.* *decoloratus* larvae collected from cattle herds in the northeastern region of the ECP.

## Materials and Method

### Ethical approval 

Before data collection, ethical approval was obtained from the University of Fort Hare’s Research Ethics Committee (Reference number: MUC021SYAW01). All experimental procedures adhered to the moral standards for experimentation established by the Society for the Prevention of Cruelty to Animals’ ethics committee on animal use.

### Study site description

This study was conducted in 33 communities within the municipalities of Elundini, Senqu, and Walter Sisulu in the ECP’s Joe Gqabi District. Elundini municipality is located at an elevation of 1,600 m above sea level. In the Southern Drakensberg Grassland, the average annual rainfall is between 800 and 1,200 mm; the average annual minimum temperature is 13°C; and the maximum temperature is 22°C. [18]. Senqu municipality is located between 1,000 and 1,500 m above sea level, with an annual average temperature of −16°C in the winter and 42°C in the summer. Montana Shrubland receives an annual rainfall between 1,000 and 1,400 mm [18]. The municipality of Walter Sisulu is located between 1,000 and 1,500 m above sea level. Under the Mixed Nama Karoo vegetation type, the annual average temperature ranges between 15°C in winter and 30°C in summer, and the annual rainfall ranges between 1,000 and 1,400 mm [18].

### Experimental animals 

Ten cattle herds were randomly selected for tick sampling at each dip tank from November 2018 to September 2019. At all times, all animals selected were over the age of 12 months and included both sexes. Cattle in the study areas were of various breeds. However, the study did not focus on the breed effect. Each locality conducted cattle dipping twice a month during the summer and once a month during the winter season. Ticks were sampled from cattle prior to dipping to ensure that the tick population was not skewed. Water supply to the dip tank has been a major issue in these areas, limiting farmers’ use of the plunge dipping system during the dry season. With a continuous grazing system, cattle rely heavily on natural pastures for feeding.

### Ticks collection and transportation for acaricide testing 

Between 0800 and 0900 h on dipping days, engorged female ticks were collected from grazing cattle; this was done because the majority of engorged ticks drop off the host in the early morning. At each dip tank, a sample of 20–30 adult female *R. decoloratus* ticks was collected from at least 10 cattle and placed in plastic collection bottles containing absorbent paper and perforated lids at a constant room temperature of 28°C and a relative humidity of >70%. Each collection bottle was labeled with the collection date, the name of the farm, and the number of cattle sampled. Ticks were immediately transported to the Acaricide Resistance Testing Laboratory at the University of the Free State’s Department of Zoology and Entomology for acaricide resistance testing. Engorged female ticks were washed on a sieve with clean tap water upon arrival at the laboratory, and all damaged and undersized ticks (weighing less than 150–350 mg) were discarded. After air-drying ticks on an absorbent paper, they were placed in a glass flask and incubated [19]. Ticks were checked daily until oviposition began. After the ticks produced their first egg at approximately +35 days, they were monitored daily for hatch date establishment, which was determined to be the day when about 70% of the larvae hatched. Then acaricide resistance testing was carried out on larvae between 15 and 21 days [19].

### Acaricides used in the study

The study tested for acaricide resistance using three dip formulations: (i) OP, (ii) synthetic pyrethroids (SPs), and (iii) amidines. These compounds were chosen because they are commonly used in South Africa, are commercially available, and have all been registered for tick control under Act 36 of 1947. For tick larvae resistance testing, the acaricide compound concentrations used are the standard recommended field concentrations for each acaricide, prepared from a 1% stock solution diluted from each acaricide group (Table 1). The concentrations used were cypermethrin 0.015 and 0.03 ppm, chlorfenvinnphos 0.03 and 0.05 ppm, and amitraz 0.025 ppm. These concentrations were prepared using double-distilled water, with one serving as a control. Ten milliliters of each concentration test and distilled water were placed in the labeled test tubes for the subsequent acaricide testing step. During the preparation process, the concentration and water were thoroughly mixed to ensure the uniformity of the acaricide solution.

### Shaw larval immersion test (SLIT)

Engorged female ticks were handled in accordance with the laboratory’s standard operating procedure (SOP) upon arrival [20]. In summary, the SOP requires that engorged females be washed on a sieve with clean tap water and that all damaged, non-engorged, and pre-laying females be discarded. Tick larvae were exposed to the field concentration of SLIT for acaricide resistance testing, and the percentage of larvae killed was used to determine efficacy. A mortality rate of at least 80% was considered adequate. In comparison, a mortality rate of less than 80% but greater than 50% was considered an indicator of resistance development, while a mortality rate of less than 50% was deemed resistant [20].

### Larvae exposure to the acaricide

The conical flask containing the larvae samples was submerged in water on a petri dish. After that, a round of filter paper with a diameter of 24 cm was placed in the stainless steel tray to absorb any water droplets that may have spilled during the start of the actual test. A foil plate comprising two circular filter papers with a diameter of approximately 11 cm was inserted into the 24 cm filter paper. The resistant test was initiated by removing the cotton stopper from the flask and inserting it into the side of the 11 cm filter paper in the pie plate using forceps. The remaining larvae in the flask’s neck were then removed with a demarcated control brush and brushed onto one sheet of filter paper, which was then covered. The flask was sealed with a cotton stopper, and the control test tubes were vortexed for 10 sec before being poured over the filter paper sandwich. After pouring the concentrations into the filter paper, the timer was started, and the procedure was repeated for 60 sec. Tick larvae were transferred to the filter paper using the uncontaminated brush. After determining the concentrations, masking tape was used to remove any larvae that had escaped into the cotton stopper. The flask was returned to the incubator box and relocated to the incubator room.

**Table 1.** Acaricide resistance test dilutions.

Dilutions made from a 30% (m/v) chlorfenvinphos solution				
Dilution number	Concentration (% m/v)	Dip	2× Distilled water (ml)	Total (ml)
Stock 1	1%	1.67 ml chlorofenvinphos solution	48.33	50
2	0.03	3 ml of stock 1	97	100
3	0.05	5 ml of stock 1	95	100
4	Control	-	10	10
**Dilutions made from a 12.5% (m/v) amitraz solution**				
Stock 1	1%	4 ml amitraz solution	46	50
2	0.03	2.5 ml of stock 1	97.5	100
3	Control	-	10	10
**Dilutions made from a 20% (m/v) cypermethrin solution**				
Stock 1	1%	1.67 ml chlorofenvinphos solution	48.33	50
2	0.03	3 ml of stock 1	97	100
3	0.05	5 ml of stock 1	95	100
4	Control	-	10	10

### Larvae postexposure to acaricide 

Using forceps, the sandwich filter paper was removed from the plate precisely 10 min after the larvae were exposed to the acaricide. A small amount of water was drained into one of the 24 cm filter paper’s corners, and the foil plate was then discarded. The sandwich filter paper was then separated and placed in the dry sections of the 24 cm filter paper to extract excess liquid. Again, the designated brush was used to transfer larvae into the pre-labeled filter paper envelope, and masking tape was used to prevent larvae from escaping through the filter paper. Two envelopes containing chemicals were then clipped together and placed in the incubator for 72 h at a temperature of ±28°C and a relative humidity greater than 70%. This procedure was repeated for each field concentration, and a new foil plate and 24 cm of paper were used for testing. Between each chemical concentration used, acetone was used to clean the trays, and separate incubator boxes were used to separate the chemical and control envelopes in the incubator room.

### Larvae mortality counting 

After 72 h, the filter paper envelopes were removed from the incubator, and all the larvae, alive and dead, were counted. Live larvae were counted on a 24 cm filter paper, taking care to avoid larvae that might flee during the counting process. The total number of live larvae was recorded in the envelope’s corner. All dead larvae were poured into the 24 cm filter paper below and counted. As a result, the mortality percentage was calculated by counting live and dead larvae. Corrected mortalities were determined using the formula described elsewhere [21]:


CM%=%i−%c100−%c×100

Where;

CM% = corrected mortality;

% *i* = % mortality in concentration; and

% *c* = % mortality in water control.

## Results

The SLIT was only carried out on *R. decoloratus* larvae as engorged *R. microplus* ticks could not meet the required sample size for resistance testing. Each resistance range was presented by its specific color, from red = indications of resistant, yellow = developing resistance, blue = effective reservation, and pink = susceptible/effective, as shown in Table 2.

### Resistance profile of amidine, OP, and SPs

Table 3 shows the defined resistance development of *R. decoloratus* larvae exposed at different field concentration levels of amidine, OP, and SPs. A total of 49% of the larvae were susceptible to amidine, with 30% developing resistance and 21% ineffective reservation at a concentration of 250 ppm. At a concentration of 300 ppm, OP displayed the greatest proportion of effective reservations (45%), with 33% being susceptible to the chemical, 18% developing resistance, and only 4% being considered resistant. On the other hand, 47% susceptibility was observed when larvae were exposed to OP at a concentration of 500 ppm, 32% showed effective reservation, and 21% indicated developing resistance. The SP results at a concentration of 150 ppm showed 44% susceptibility, 37% effective reservation, 12% developing resistance, and 7% resistance. However, on the other hand, SPs at a concentration of 300 ppm recorded the most significant proportion of effective reservations (34%), followed by developing resistance (30%), with 23% of the samples susceptible and 13% resistant.

**Figure 1.**
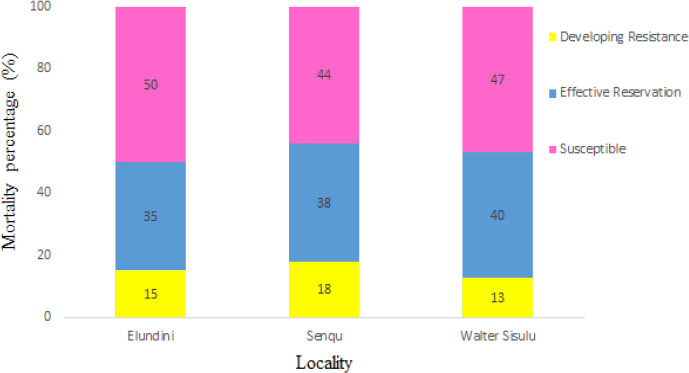
Resista~nce profiles of R. decoloratus larvae exposed to amidine.

**Table 2.** The range of resistance percentages used to display the larvae resistance.

Mortality count range (%)	Color	Result interpretation
0%<50%	Red	Indications of resistant
50%<80%	Yellow	Developing resistance
80%<90%	Blue	Effective reservation
90%–100%	Pink	Susceptible

**Table 3.** Resistance status of amidine, OP, and SPs used at 150 ppm, 250 ppm, 300 ppm and 500 ppm field concentration levels.

		Field concentration levels			
	Resistant test	150 ppm	250 ppm	300 ppm	500 ppm
	Developing resistance	-	21%	-	-
Amidine	Effective reservation	-	30%	-	-
	Susceptible	-	49%	-	-
	Resistant	-	-	4%	-
OP	Developing resistance	-	-	18%	21%
	Effective reservation	-	-	45%	32%
	Susceptible	-	-	33%	47%
	Resistant	7%	-	13%	-
Pyrethroids	Developing resistance	12%	-	30%	-
	Effective reservation	37%	-	34%	-
	Susceptible	44%	-	23%	-

### Rhiphicephalus decoloratus larvae resistance profiles exposed to amidine

Figure 1 shows the mortality counts of *R. decoloratus* tick’s larvae collected from Elundini, Senqu, and Walter Sisulu exposed to amidine field concentration. Ticks collected from Elundini, Walter Sisulu, and Senqu were susceptible to amidine and showed 50%, 47%, and 44% mortality counts, respectively. More so, mortality counts for effective reservation were observed mainly in Walter Sisulu (40%), 38% at Senqu, and the lowest mortality counts at the Elundini region (35%). Resistance development of the larvae to the chemical was extensively observed in Senqu (18%), Elundini (15%), and Walter Sisulu (13%) regions. Ticks did not show resistance when exposed to this chemical across the localities.

### Rhiphicephalus decoloratus larvae resistance profiles exposed to OP

**Figure 2.**
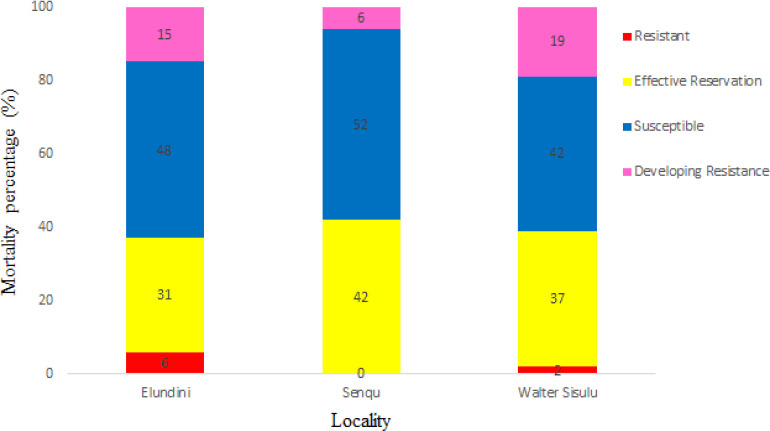
Resistance profiles of R. decoloratus larvae exposed to OP.

Figure 2 shows the mortality counts of *R. decoloratus* tick’s larvae collected from Elundini, Senqu, and Walter Sisulu exposed to OP field concentration. Susceptible mortality counts were primarily observed in Senqu (52%), Elundini (48%), and Walter Sisulu (42%). The highest mortality counts for effective reservations were recorded in Senqu (42%), Walter Sisulu (37%), and Elundini (31%). The highest larvae within the resistance development range to the chemical were found in Walter Sisulu (19%), followed by Elundini (15%), and the lowest counts (6%) in the Senqu region. 6% and 2% mortality counts were observed in the Elundini and Walter Sisulu regions, respectively. There was no tick resistance to OP in the Senqu region.

### Rhiphicephalus decoloratus larvae resistance profiles exposed to SPs

**Figure 3.**
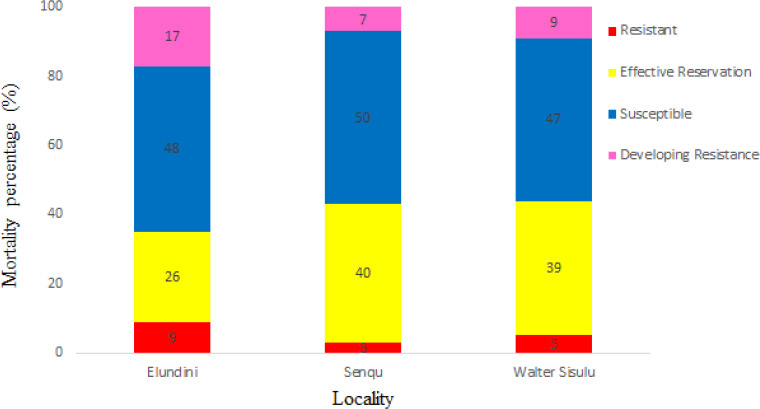
Resistance profiles of R. decoloratus larvae exposed to SPs.

Figure 3 shows the mortality counts of *R. decoloratus* tick’s larvae collected from Elundini, Senqu, and Walter Sisulu exposed to SP concentrations. Larvae mortality counts were susceptible in Senqu (50%), Elundini (48%), and Walter Sisulu (47%), respectively. Similarly, larvae mortality counts in the effective reservation category were 40% in Senqu, 39% in Walter Sisulu, and the lowest counts in Elundini (26%). The highest larvae within the resistance development range to the chemical were observed in Elundini (17%), Walter Sisulu (9%), and Senqu (7%). On the other hand, the greatest mortality counts of larvae described as resistant to the applied chemical were observed mainly in Elundini (9%), Walter Sisulu (5%), and Senqu had the lowest counts (3%) compared to the other localities.

## Discussion

Tick infestation is the most economically important ectoparasite of livestock and has been reported globally [22]. Many South African studies [23–26,17,27–30] have documented studies of *R. microplus* resistance to the most commonly used commercial acaricides for tick control. The common acaricides used for the management of ticks include formamidines, phenylpyrazoles, macrocyclic lactones, SPs, and OPs. The indiscriminate use and misapplication of acaricides has escalated selection pressure for tick resistance to acaricides [31]. Studies have further reported the displacement of the African blue tick (*R. decoloratus*) by the Asian cattle blue tick (*R. microplus*). However, no such displacement was observed in the current study. The present study found patchy engorged *R. microplus* tick specimens in each study site. Similar findings were also reported by Pottinger [32], who reported fewer *R. microplus* counts in the study conducted in the coastal regions of the ECP. The collected *R. microplus* samples did not meet the sample size for resistance testing. These findings were attributed to the susceptibility of *R. microplus* to currently used acaricides in the study localities. Also, the resistance information of *R. decoloratus *in South Africa, particularly in the ECP, is mostly outdated as more focus has been shifted toward the invasive tick species, *R. microplus* [33,8,34,9].

Over the past 10 years, the three acaricide compounds (amidine, OP, and SPs)have been used to control ticks as they are known for their low toxicity to cattle and other animal species on which ticks feed. This chemical acts as an octopamine receptor when applied to the tick, leading to reduced numbers of active neurons, resulting in tick paralysis and death [3]. The mechanism of tick resistance to acaricide is described by an increase in the tick’s metabolic activity that produces metabolic enzymes that detoxify any toxic substance as soon as possible before it gets to the target sites [35]. The current study found that the majority of the ticks were susceptible to exposure to different field-level concentrations of the acaricide amidines (49% at 250 ppm), OPs (33% and 47% at 300 ppm and 500 ppm, respectively), and SPs (44% and 23% at 150 ppm and 300 ppm, respectively). Tick susceptibility may be responsible for the low tick counts in the study areas, particularly the blue tick. The low tick count may also be linked to the prolonged drought in the ECP over the past 5 years, which has limited adequate tick habitat for an increasing population [36].

The current study reported *R. decoloratu*s larvae resistance when exposed to OP and SPs, with no resistance reported to amidines. These findings concur with the report conducted elsewhere [34,30]. They found similar results where all the tick larvae did not show resistance during exposure to the amidines. It was further argued that even though the three acaricide groups have been used over the years, amidines have not been commonly used in high tick areas, which lowers the chances for tick selection pressure on amidines compared with the OPs and SPs [37,30]. Hence, it is further anticipated that amidines effectively control single and multi-host ticks [38].

Of the three localities, except for Senqu, the tested larvae did not show resistance when exposed to all the acaricides. This was attributed to the fact that there was a well-developed tick control program at Senqu. In these regions, it was compulsory for every cattle farmer to bring the cattle for dipping. This action suppressed the tick population in the region and subsequently made it difficult for ticks to develop resistance. The majority of the cattle farmers indicated that they increase acaricide concentration during the high tick season, resulting in complete mortalities of ticks after acaricide application. The only worry seemed to be the presence of acaricidal residues [11]. Among other tick resistance factors, acaricide use over a long period has been the major contributing factor toward the larvae’s resistance to OPs and SPs [39,8]. However, even though OPs and SPs showed resistance, findings from the studies conducted earlier [9] and [7] suggested that the two acaricides effectively decrease tick populations when applied at an effective therapeutic concentration. Thus, the emergence of acaricide resistance in *R. decoloratus* ticks should motivate tick control programs in the regions to mitigate the chances of complete resistance to the commonly used chemicals [40].

## Conclusion

This study documented *R. decoloratus* larvae’s resistance to OP and SPs except for amidines. This is an important finding given the regular use of OP and SPs in the management of ticks in South Africa. We further observed that amidine acaricides were the most effective for controlling *R. decoloratus* larvae. Thus, this study recommends that future acaricide application strategies incorporate knowledge of tick dynamics, such as available tick species, as the tick population differs from region to region based on host availability and vegetation. Moreover, our study recommends that acaricides be diluted by trained personnel and guided by the manufacturers’ recommendations for effectiveness. Also, the rotation of the acaricides should be practiced using different modes of action.

## List of Abbreviations

BHC, Benzene hexachloride; cm, Centimeter; ECP, Eastern Cape Province; h, hour; LPT, Larval packed test; LTT, Larval tarsal test; mm, Millimeter; ppm, Parts per million; SLIT, Shaw larval immersion test; SOP, Standard operating procedure.
